# A preliminary analysis of volatile metabolites of human induced pluripotent stem cells along the *in vitro* differentiation

**DOI:** 10.1038/s41598-017-01790-5

**Published:** 2017-05-09

**Authors:** Rosamaria Capuano, Paola Spitalieri, Rosa Valentina Talarico, Ana Carolina Domakoski, Alexandro Catini, Roberto Paolesse, Eugenio Martinelli, Giuseppe Novelli, Federica Sangiuolo, Corrado Di Natale

**Affiliations:** 1 Department of Electronic Engineering, University of Rome Tor Vergata, Via del Politecnico 1, 00133 Rome, Italy; 2 Department of Biomedicine and Prevention, University of Rome Tor Vergata, Via Montpellier 1, 00133 Rome, Italy; 3 Department of Chemical Science and Technology, University of Rome Tor Vergata, Via della Ricerca Scientifica, 00133 Rome, Italy

## Abstract

Cellular metabolism of stem cell biology is still an unexplored field. However, considering the amount of information carried by metabolomes, this is a promising field for a fast identification of stem cells itself and during the differentiation process. One of the goals of such application is the identification of residual pluripotent cells before cell transplantation to avoid the occurrence of teratomas. In this paper, we investigated *in vitro* the volatile compounds (VOCs) released during human induced pluripotent stem cells (hiPSCs) reprogramming. In particular, we studied hiPSCs differentiation to floating and adherent embryoid bodies until early neural progenitor cells. A preliminary Gas Chromatographic/Mass Spectrometer (GC/MS) analysis, based on a single extraction method and chromatographic separation, indicated 17 volatile compounds whose relative abundance is altered in each step of the differentiation process. The pattern of VOCs shown by hiPSCs is well distinct and makes these cells sharply separated from the other steps of differentiations. Similar behaviour has also been observed with an array of metalloporphyrins based gas sensors. The use of electronic sensors to control the process of differentiation of pluripotent stem cells might suggest a novel perspective for a fast and on-line control of differentiation processes.

## Introduction

Human induced pluripotent stem cells (hiPSCs) are widely used as an ideal target for disease modeling and drug discovery as well as for planning autologous cell-based therapy for genetic and degenerative diseases^1, 2^. When derived from patient cells, hiPSCs represent an available model system for studying the pathogenic mechanisms and the progression of diseases, particularly in those cases where animal models do not exactly reproduce the human phenotype or when disease-target cells types are simply not available. Each cell can be reprogrammed to hiPSCs, *in vitro* corrected, if necessary, and transplanted back to the patient after differentiating them into one or more cell types phenotypically developing the disease of interest. The overall aim is to recover the target-damaged tissue, avoiding any immune response. Recently extra-embryonic tissues such as chorionic villus (CV) and amniotic fluid (AF) have been successfully reprogrammed into hiPSCs^3^. Nevertheless, when inoculated *in vivo*, if not fully differentiated, hiPSCs can generate teratomas due to the characteristic of the residual pluripotent stem cells^4^.

A number of approaches has been considered to eliminate the risk of teratoma formation from undifferentiated hiPSCs. Some of these methods aim at the selective segregation of differentiated cells and then to the suppression of the residual undifferentiated cells^5^. The fully differentiation of hiPSCs has then to be confirmed by some specific method, targeting or the surface proteins or the genome^6^. Recently, the measure of the electrochemical potential was proposed for the detection of hiPSCs^7^.

Cellular metabolism is an interesting alternative to study stem cells during the various steps of differentiation, evidencing any changes due to experimental conditions. On these bases, it is reasonable to suppose that the transition of stem cells from pluripotency to the complete differentiation, might give rise to a dramatic change of cellular metabolism. First evidences of metabolic differences between induced pluripotent stem cells, parental fibroblasts, and embryonic stem cells have been provided^8^.

Within the metabolic products, the volatile organic compounds (VOCs) are attracting interest for the supposed simplicity of their collection, the intrinsic non-invasiveness and the wide availability of the analysis methods^9^. To this regard, several studies show that the headspace of cancer cells exhibit a VOCs profile which is altered as compared to that of normal cells^10, 11^. The analytical chemistry approach to the measure of the volatile fraction of the metabolome (sometimes called *volatilome*) is based on the use of instruments that operate a separation of the gaseous sample into its molecular components. The separation is paralleled by the sequential detection and characterization of each component. Gas Chromatography/Mass-Spectrometer (GC/MS) is the standard approach because it is able to provide reproducible retention characteristics and mass spectra, high sensitivity, and the availability of a wide range of libraries for the identification of compounds^12^. More sophisticated techniques, such as Secondary ElectroSpray Ionization^13^ and Proton Transfer Reaction-Mass Spectrometry^14^ to mention few, are also available for more detailed investigation of the volatilome.

In the last decade, the analytical chemistry approach has been complemented by the arrays of partially selective gas sensors^15^. These are ensembles of sensors each characterized by the sensitivity to a large variety of compounds. Since individual sensors are not selective, the total volatilome is converted in a fingerprint of sensor responses. Fingerprints are then processed by some multivariate statistical classifier in order to provide the identification of classes. This process is based on a combinatorial selectivity procedure analogous to that found in olfaction^16^ and for this reason these instruments are widely known as “electronic noses”.

Among the other applications, electronic noses have been successfully used for cell headspace analysis, for instance to identify lung^17^ and breast cancer cells^18^.

In this study we combine Solid-Phase Microextraction/Gas Chromatography/Mass Spectrometry (SPME/GC/MS) and the electronic nose to investigate the modification of volatile metabolites in the cell culture headspace during cell reprogramming from chorionic villus samples (CVS) to hiPSCs and successively during their differentiation process through the embryoid bodies (EBs) formation. The used electronic nose is an array of metalloporphyrins coated quartz microbalances (QMBs) of the same kind of those previously used for lung cancer detection from breath analysis^19, 20^.

Although the investigation has been limited by the proper selectivity of the SPME/GC/MS equipment, the results show that each step is characterized by a proper profile of volatile metabolites and that the electronic nose can capture these differences, making identifiable each step of the process.

This study suggests a new perspective for monitoring the presence of residual undifferentiated cells during and after an *in vitro* differentiation protocol. This aspect, when further reinforced by extended studies based on a more comprehensive analytical tools, would represent a huge significance in regenerative protocols, avoiding the risk of teratoma formation after *in vivo* transplantation of committed hiPSCs cells.

## Results

**Figure 1:**
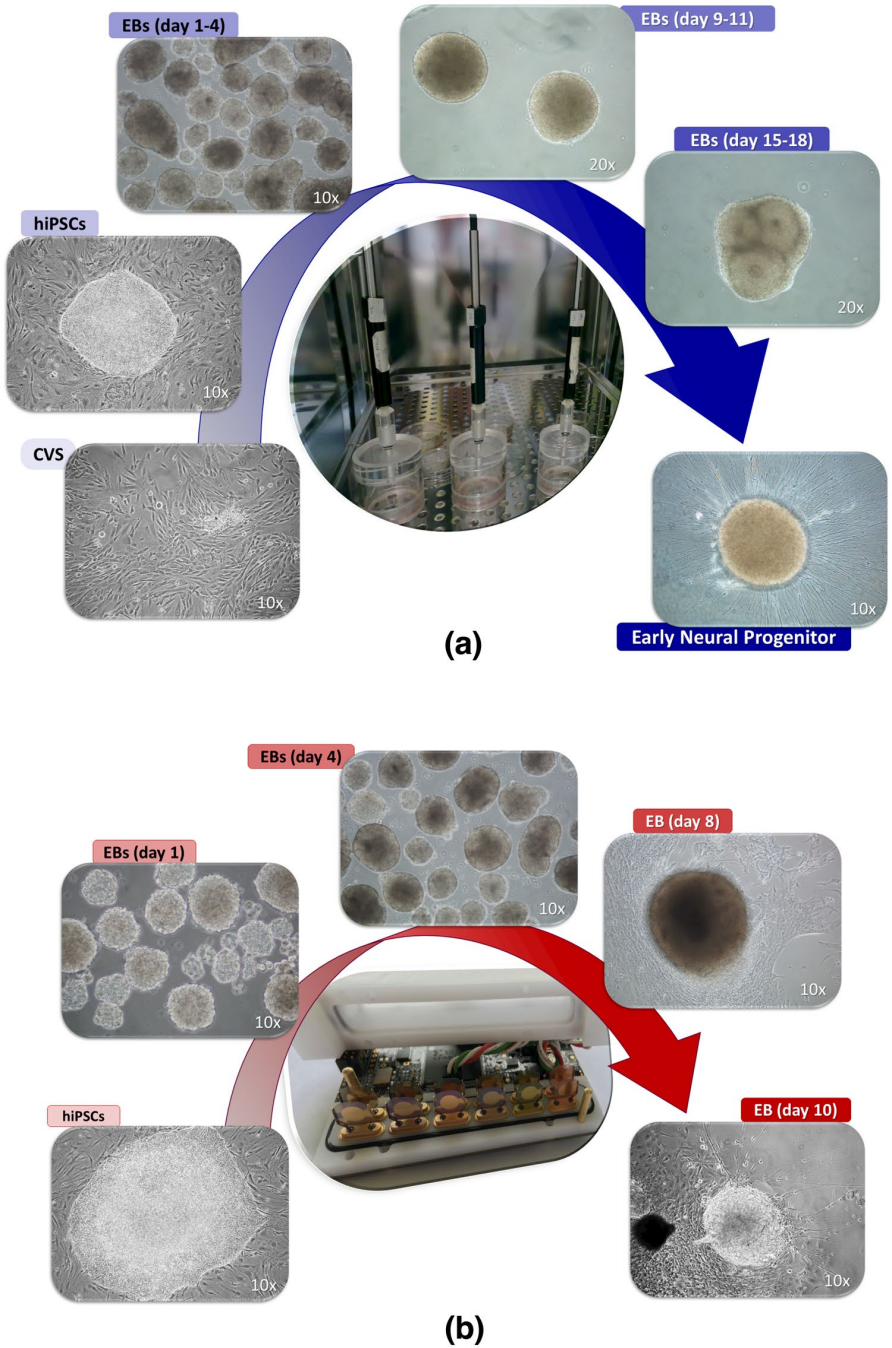
(**a**) Samples analysed by GC/MS. Chorionic villus samples (CVS) were used to obtain human induced pluripotent stem cells (hiPSCs), that were successively differentiated into early neural progenitors (NPs) through the formation of floating embryoid bodies at different days of differentiation (floating EBs days 1–4, floating EBs days 9–11, floating EBs days 15–18). (**b**) Samples analysed by electronic nose. Human induced pluripotent stem cells (hiPSCs) were spontaneously differentiated into mesodermal, ectodermal and endodermal germ layers (adherent EBs day 8 and day 10) through the formation of floating embryoid bodies at different days of differentiation (floating EBs day 1 and floating EBs day 4).

In this work, the VOCs analysis was carried out with two instruments: the GC/MS and the electronics nose. These analyses were aimed first at identifying the VOCs and then at evaluating the qualitative differences between the samples. GC/MS analyzed the samples showed in Fig. 1a: human induced pluripotent stem cells (hiPSCs) reprogrammed from chorionic villus samples (CVSs) and successively differentiated into early neural progenitors (NPs) through the formation of embryoid bodies (EBs).

Figure 1b shows the samples that have been analyzed with the electronic nose: human induced pluripotent stem cells (hiPSCs) spontaneously differentiated into the three germ layers (EBs day 8 and EBs day 10) through the formation of floating embryoid bodies (EBs day 1 and EBs day 4).

### Gas Chromatography/Mass Spectrometer

GC/MS analysis has been applied to measure the VOCs in headspace of the cells shown in Fig. 1. The collection of VOCs has been carried out with the Solid Phase MicroExtraction (SPME) technique using a general purpose fiber. It is important to point out that the selectivity of the SPME fibre and the GC/MS colums restrict the investigation to a limited set of compounds.

The complete list of the identified VOCs is found in Table S2 in the Supplementary Information file. These compounds are supposed to originate from both the background culture media and the metabolic activity of the cells. The identity of those compounds which are commercially available have been validated by a comparison of the elution time and the mass spectra in samples with that of standard compounds added to the culture media and measured with the same procedure used for the samples. The rest of compounds are putatively identified by a comparison of mass spectra with the NIST libraries. The percentage of identification score is reported, for these compounds, in Table S2.

Statistical significance of the relative abundances of VOCs among the cell lines have been determined with a non-parametric Kruskal-Wallis rank sum test. The results are expressed as the null hypothesis probability (p value). The same analysis has also been conducted on VOCs released by the different culture medium in order to eliminate confounding VOCs which are due to the different substrates rather then to the cells metabolism.

In the ANOVA analysis the floating EBs at days 1–4 and days 9–11 were merged in a single class.

Due to the different medium used for stem cell growth, each condition was individually measured, in order to separate the compounds specifically released by cells. Moreover, each measure has been replicated almost three times in independent samples, that means in biological replicates derived from different individuals.

The influence of ageing processes in culture media have been analyzed measuring the volatile compounds after 1 h and 24 h in the incubator at 5% CO_2_ at 37 °C. As comparison, also deionized water was measured in the same conditions. Culture media and water did not show any appreciable change in the volatile compounds.

In order to eliminate the influence of culture media, the compounds statistically different among pure culture medium have been removed from the successive analysis.

On the other hand, it has to be considered that the culture media may also indirectly influence the metabolic products of the cellular cultures and then, from a very restrictive point of view, the segregation of the specific cell VOCs from the culture background is not straightforward.

Table 1 lists all the compounds whose statistical distribution of the relative abundance is significantly different (p < 0.05) in binary comparisons among different types of cells. In detail, Table 1 shows the p-values related to the comparison between chorionic villus samples (CVS) and hiPSCs, hiPSCs and floating embryoid bodies at three different culture time points (1–4 days, 9–11 days, and 15–18 days), CVS and early neural progenitors (NPs), hiPSCs and early neural progenitors (NPs), and finally the three stages of floating embryoid bodies (1–4 days, 9–11 days, 15–18 days) and early neural progenitors (NPs).

**Table 1:** List of volatile compounds whose statistical distribution is significantly differently distributed in the binay comparison between the kinds of cells.

Retention time	Name		CVS/hiPSCs	hiPSCs/EBs 1–4 days	hiPSCs/EBs 9–11 days	hiPSCs/EBs 15/18 days	CVS/EBs 1–4 days	CVS/EB 9–11 days	CVS/EB 15–18 days	CVS/early NPs	hiPSCs/early NPs	EBs 1–4 days/early NPs	EBs 9–11 days/early NPs	EBs 15–18 days/early NPs
6.725	Hexanal	Identifed										0.03		0.007
9.270	3-Hexen-1-ol, propanoate, (Z)-	Identifed	0.01				0.02	0.02	0.02					
9.486	Styrene	Identifed				<0.0001			<0.0001	0.001	0.0014	0.001	0.001	<0.0001
12.362	Ethanone, 1-cyclopropyl-2-(4-pyridinyl)−	Putative				<0.0001			0.003					0.03
13.34	1-Hexanol, 2-ethyl-	Identifed	<0.0001	0.003	0.001	0.002	<0.0001	<0.0001	<0.0001	<0.0001				0.04
15.178	Nonanal	Identifed		0.02						0.006	<0.0001			0.04
15.474	Ethanone, 2,2′-(octahydro-2,3-quinoxalinediylidene)bis[1-phenyl-	Putative		0.004	0.03	0.04					<0.0001			0.02
17.404	Tridecane	Identifed										0.005		
17.469	Decanal	Identifed								0.01	0.041		0.04	
20.958	Butanoic acid, 2-methylpropyl ester	Putative									0.041			
23.400	Phenol, 3,5-bis (1,1-dimethylethyl)-	Putative					<0.0001	<0.0001	<0.0001		<0.0001			
24.010	Propanoic acid, 2-methyl−, anhydride	Putative									0.008			
24.967	hexadecane	Identifed				<0.0001				<0.0001				
26.609	Dodecane, 4,6-dimethyl-	Putative		0.04	0.01	0.01	0.04	0.03	0.02	0.0008	<0.0001	<0.0001		
28.161	Heptadecane	Identifed		<0.0001	<0.0001	<0.0001	0.03	0.02	0.01	0.001	<0.0001			
28.310	3,5-Dimethyl-4-octanone	Identifed								0.007	0.03			

p-values are calculated from a non-parametric Kruskal-Wallis rank sum test.

GC/MS analysis indicates that compounds making the difference between the investigated cell lines exist for all the binary separations. Some compounds are discriminant in more than one case, for instance 1-hexanol-2-ethyl is an almost ubiquitous indicator in all binary comparisons, except when floating EBs are compared to early NP cells where it appears, with a larger p-value, after two weeks of incubation. Other recurrent compounds are found between CVS, hiPSCs, and EBs, while different compounds emerge when early NPs are considered. Specifically, the relative abundance of decanal, Butanoic acid, 2-methylpropyl ester, Propanoic acid, 2-methyl-, anhydride, 3-Hexanone, 2,4-dimethyl and 3,5-Dimethyl-4-octanone is significantly different only when hiPSCs are compared to early NPs.

Some of these VOCs have been previously observed in the headspace of cultured cells, tissues and organic fluids. Anomalous concentrations of 1-hexanol, 2-ethyl have been found in the headspace lung cancer pleural effusion^21^ and lung cancer cell line^22^. Aldehydes, such as nonanal, and decanal, have been observed in excess in the breath of lung cancer affected individuals^23^, in the headspace of lung cancer cells^24^ and in human epithelial cervical carcinoma cells^25^. Furthermore, decanal has been suggested to be associated to gene mutations in lung cancer^26^. Styrene is another recurrent compound in Table 1, and its presence in the breath has been associated to lung cancer^27^. Combination of styrene, decanal and nonanal was identified in the breath of ovarian cancer affected individuals^28^.

The relative abundance of all the compounds selected by the ANOVA analysis and listed in Table 1, have been assembled to form a pattern of cells headspace. Then, a simple comparison among the different cell lines can be obtained by a multivariate representation of the set of patterns using the principal component analysis (PCA). PCA is a popular method in multivariate analysis to represent multidimensional data. PCA decomposes the set of multivariate data into non-correlated variables^29^. In practice, PCA introduces a number of novel variables which are linear combinations of the pattern elements. Since the principal components are not correlated, the total variance of the data set is simply the sum of the variance explained in each principal component. The carried variance establishes a hierarchy among the principal components, with the straightforward assumption that the first principal components carrying the largest variance are representative of the collective behavior of the pattern’s elements.

**Figure 2:**
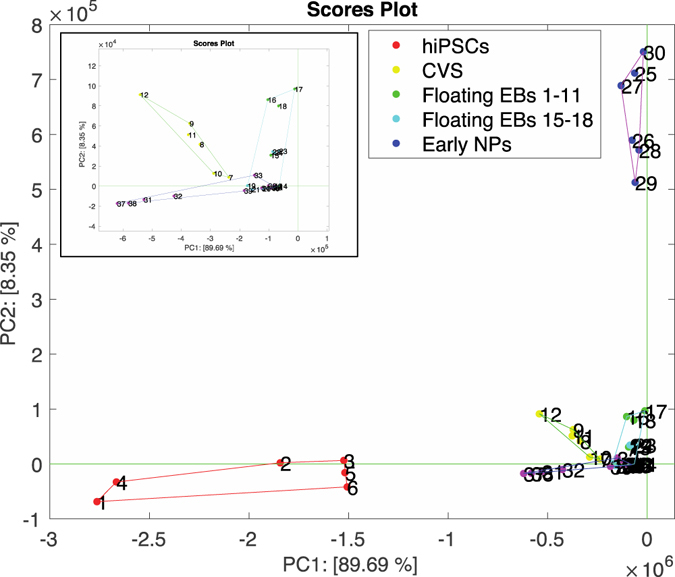
Plot of the first two principal components of the GC-MS data matrix considering all the compounds listed in Table 1. The inset shows the magnified view of the data around the origin. Floating EBs at 1–4 days and EBs at 9–11 days were merged in one sample because showing an overlapping profile.

PCA has been calculated on the pool of VOCs identified by the binary ANOVA discussed above, data have not been scaled or normalized in order to preserve the information about the abundance of the single compounds. Figure 2 shows the plot of the first two principal components of the GC/MS patterns. The most evident feature of the plot is the large separation among hiPSCs, floating EBs at day 15–18 and the other samples. Specifically, except hiPSCs, floating EBs at day 15–18 all data are clustered together. A magnified view of these data shows the tendency to discriminate also among CVS, floating EBs at day 1–11 of differentiation and early NPs.

Besides to represent the samples, PCA allows to evaluate the contribution of each element of the pattern to the multivariate representation. The contributions of the pattern elements are called loadings. The analysis of the loadings of the PCA of the GC/MS patterns (See Fig. S3 in the Supplementary File) shows that the sharp separation of hiPSCs and floating EBs at day 1–11 is due to the excess of relative abundance of 1-hexanol-2-ethyl and styrene respectively. A minor role in the separation of hiPSCs is played by Dodecane, 4,6-dimethyl- and 3,5-Dimethyl-4-octanone. While hexanal and Ethanone, 1-cyclopropyl-2-(4-pyridinyl)- support the separation of floating EBs at day 1–11.

As mentioned above, styrene and 1-hexanol-2-ethyl are among the key compounds to discriminate between hiPSC and EBs. These compounds are also the most ubiquituous among all the identified compounds since they have been found in all samples including the culture media. The relationship between these compounds and the discrimination of samples has been based on the relative abundance whose behaviour may be not coincident with the actual concentration. To elucidate the role of styrene and 1-hexanol-2-ethyl the concentration of these compounds in the measured samples have been estimated. For the scope an external calibration procedure has been used adding known concentration of these compounds to the culture media and measuring the volatile compounds with the same procedure used for the samples. The concentrations are found in the range of ppbv.

The concentration of styrene and 1-hexanol-2-ethyl was previously quantified in the headspace of lung cancer cells. The concentration of styrene is in agreement with previous findings^30^, but the concentration of 1-hexanol-2-ethyl in all samples is about two orders of magnitude smaller than that released by lung cancer cells^22^.

**Figure 3:**
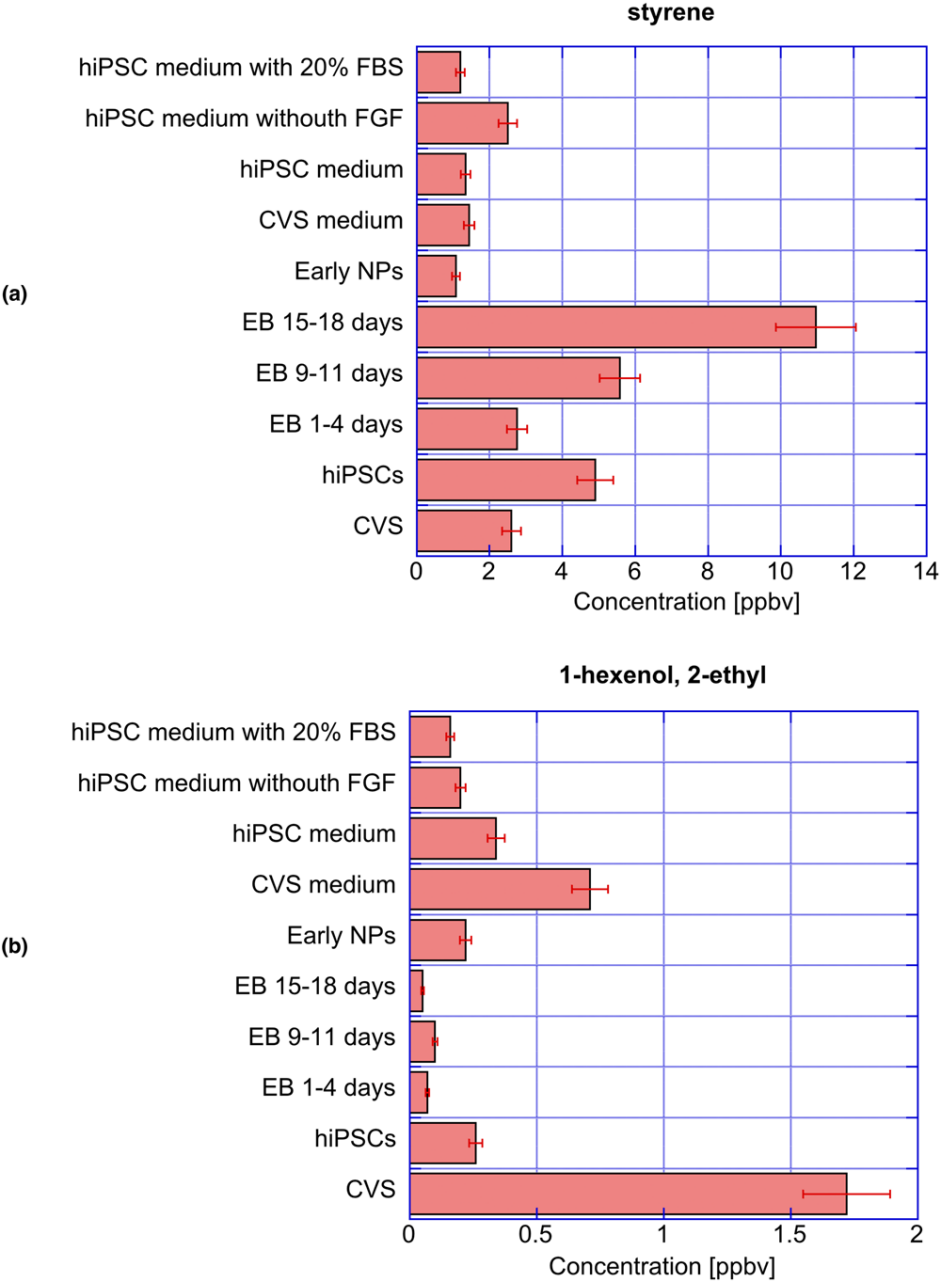
Concentration of styrene (**a**) and 1-hexenol-2-ethyl (**b**) in the analysed samples.

Figure 3 shows the behaviour of styrene and 1-hexenol-2-ethyl concentration. The presence of the two compounds in cells samples respect to the culture media is opposite. Styrene is more abundant in cells while 1-hexenol-2-ethyl is more abundant in culture media with the noteworthy exception of CVS.

For a correct evaluation of the concentration behaviour, the amount of cells in each sample have been considered. Three cultures were examined for each kind of cell, and the averages are listed in Table 2. The amount of cells scarcely affects the concentration of styrene and 1-hexanol-2-ethyl (see Fig. S4) which is then likely due to the metabolic processes in cells. In particular the large concentration of 1-hexanol-2-ethyl in CVS is not explained by a mere concentration effect.

**Table 2:** Cell counts in the studied samples. Each value is the average of counts in three samples derived from different individuals.

CVS	hiPSCs	EBs 1–4 days	EBs 9–11 days	EBs 15–18 days	early NPs
120 10^3^ cells/cm^2^	200 10^3^ cells/cm^2^	95 10^3^ cells/cm^2^	142 10^3^ cells/cm^2^	166 10^3^ cells/cm^2^	142 10^3^ cells/cm^2^
Growth area: 21 cm^2^	Growth area: 21 cm^2^	Growth area: 21 cm^2^	Growth area: 21 cm^2^	Growth area: 21 cm^2^	Growth area: 21 cm^2^
Total number of cells: 2.5 10^6^	Total number of cells: 4 10^6^	Total number of cells: 2 10^6^	Total number of cells: 3 10^6^	Total number of cells: 3.5 10^6^	Total number of cells: 3 10^6^

### Electronic nose

**Figure 4:**
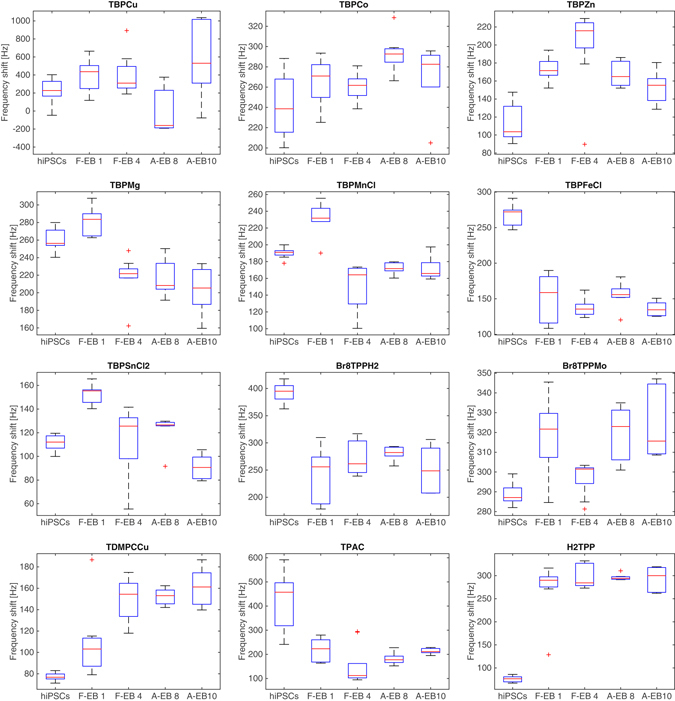
Statistical distribution of the sensors signals among the different classes of cells. The distribution is represented as a box-plot where the mean (red line), the standard deviations (the extremities of the box) and the outliers (crosses) are given for each class. Classes acronyms have been used: F-EB 1: floating EB day 1; F-EB 2: floating EB day 2; A-EB 8: EB in adhesion day 8; A-EB10: EB in adhesion day 10.

The electronic nose measured the total headspace of hiPSCs, floating EBs at day 1 and day 4, and the three germ layers corresponding to EBs in adhesion at day 8 and day 10 (see Fig. 1b). This experiment was aimed at understanding if the differences found with the GC/MS could actually be captured by an array of sensors where neither separation nor selection among the compounds can be operated but rather the totality of the headspace is simultaneously presented to the sensors. The statistic distribution of the signals of individual sensors in each kind of sample is shown in Fig. 4.

The behavior of sensor signals illustrates the combinatorial principle of the sensor array. Except sensors TBPCu, TBPCo, and Br_8_TPPMo, which are less sensitive to the differentiation process, all the other sensors display a clear relationship with the sequential process. TDMPCCu, and H_2_TPP show an increase of the signal with the progress of the process. TBPZn, TBPMnCl, and TBPSnCl2 present the largest response for the floating EB either at 1or 4 day. Finally, all the others show a signal which decreases as hiPSCs evolves into the differentiated form.

**Figure 5:**
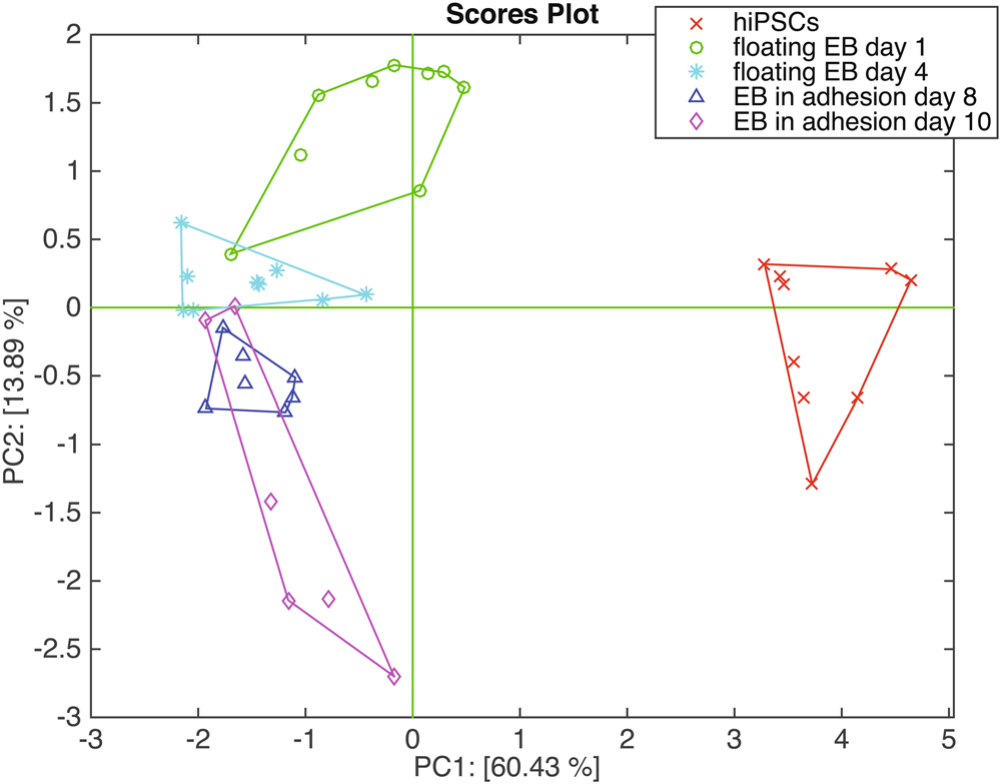
Plot of the first two principal components of the data matrix of the sensor array related to the cell headspace.

Although some of the individual sensors (H_2_TPP, TBPFeCl, and Br_8_TPPH_2_) can segregate hiPSCs from the rest of cells, the multivariate analysis of whole sensor array is the most proper method to capture the global discrimination capabilities. Figure 5 shows the plot of the first two principal components. PCA has been calculated on auto-scaled data namely the data have been normalized to zero mean and unitary variance.

The net separation of hiPSCs with respect to the other cells is largely expected after considering the individual sensors signals shown in Fig. 4. Adherent EBs (day 8 and day 10) and floating EBs are separated, even if, with a minor gap between them. Interestingly, the sensor array captures also the variations due to the increase of the culture time of EBs in suspension (among floating EBs at 1 and 4 days) while for the EBs in adhesion there is not a evident changes between 8 and 10 days. The first two principal components account for about 73% of the total variance of the data. Noteworthy, no additional separation among the sets is observed increasing the number of principal components. This suggests that sensors may be rather sensitive to other variables (e.g. humidity and temperature). Samples randomized sequence avoided that these variables co-variated with the classes of cells and influence the results.

The exiguity of the data set do not allow for the development of a properly validated classification model using independent training and test sets. However, a partial least squares discriminant analysis (PLSDA) model has been calculated in order to demonstrate the statistical discrimination of hiPSCs, floating EB, and EB in adhesion^31^. The model has been cross-validated with a leave-one-out approach and it shows, as expected from PCA, a complete separation of hiPSCs respect to the EBs and an almost complete separation of floating EB from EB in adhesion with a total classification rate of 92.5%.

A permutation test has been performed calculating the classification rate of the PLSDA model for random class membership permutations. The classification related to the separation of hiPSCs, floating EB, and EB in adhesion lies beyond the 99% of confidence bound of the classification rates distribution^32^. Details about the classification model, the ROC curves and the results of the permutation test are found in the Supplementary Information File.

**Figure 6:**
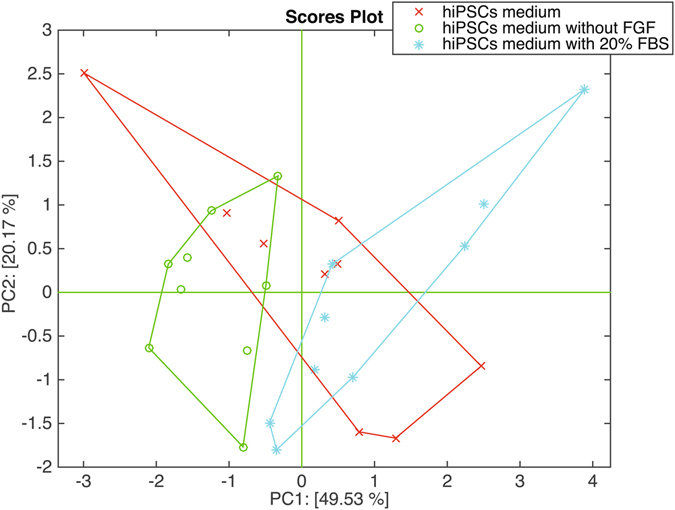
Plot of the first two principal components of the data matrix of the sensor array related to the cell culture media background.

In GC/MS data analysis the influence of the background culture media has been eliminated excluding from the analysis those compounds whose abundance depends on the background. In case of sensors, since these devices measure the total volatile compounds without separating them in individual components, the influence of the background cannot be easily segregated. The culture media backgrounds have been separately measured without cells. Figure 6 shows the first two principal components of the data related to the three culture media related to the cells analyzed with the electronic nose. In this case, a separation is observed between the culture media of floating EBs (hiPSCs medium without FGF), and spontaneously differentiated EBs in adhesion (EBs medium with 20% FBS), while the hiPSCs medium is overlapped with both. Since the sensor array cannot discriminate among the culture media, we can conclude that the observed differences among the cell classes are due to the different cellular metabolic products. Furthermore, a PCA plot of cells and culture media shows the departure of cell samples from their respective culture media (see Fig. S8).

## Discussion

hiPSCs represent a promising tool in regenerative medicine but, at the same time, their self -renewal and pluripotency capacities endow them with a strong tumorigenic potential^33^.

hiPSCs tumorigenicity can be divided into two separate categories: malignant transformation of differentiated hiPSCs and benign teratoma formation from residual undifferentiated hiPSCs, either of which can produce tumors consisting of either one or all three germ layers, respectively^4^. The maintenance of pluripotency is based on several interconnected gene expression networks involved in gene repair, cell proliferation and checkpoint in oncogenesis too. The central nature of such oncogenic properties is so fundamental to hiPSC identity that teratoma formation represents a gold standard for demonstration of their pluripotency. Moreover oncogenic risk is also associated with pluripotency induction^34^, as well as also the prolonged time of culture *in vitro* ^35^. Thus removal, before transplantation, of tumorigenic cells is preferable. Several strategies have been implemented in order to develop the necessary stringent manufacturing practices and high levels of quality control are strictly focused to eliminate residual undifferentiated cells before transplantation.

In this paper, we studied the relationship between VOCs profile and the hiPSCs along the successive steps of differentiation. Results support the hypothesis that the volatile fraction of the metabolic profile changes along the differentiation process as a reflection of the dramatic variations occurring in the cells.

GC/MS analysis evidences a number of compounds whose relative abundance can signal the difference between the various phases of the differentiation. Most of the compounds are aldehydes, alcohols, and alkanes. All these VOCs are typical of human fluids and breath. It is interesting to note that hexanal and styrene have been identified among the few compounds that are ubiquitous in the body^36^. It is worth to remark that these compounds have been detected thanks to their affinity with the SPME and the GC/MS column. As a consequence, this study cannot exclude that additional and even more discriminating volatile compounds might be found using different experimental setups.

In these experiments, the sharp separation of hiPSCs and floating EBs at day 1–11 in the PCA scores plot in Fig. 3 is largely due to the variable relative abundance of 1-Hexanol-2-ethyl- for floating EBs at day 1–11 and styrene for hiPSCs.

The role of styrene and 1-hexenol-2-ethyl is intriguing because at the same time they makes the difference among samples, and are found in all samples including the culture media. Quantitative analysis shown an opposite behaviour. Styrene is more abundant in cells than in culture media, but 1-hexenol-2-ethyl is more abundant in culture media than cells. Besides these differences, both the compounds show a certain correlation with the differentiation steps. For instance, styrene in EBs increases with the days and show a net drop in the early NPs.

It is also interesting to note the peculiar abundance of 1-hexenol-2-ethyl in CVS which is the only case where the concentration in cells exceeds the culture media. Moreover, it is interesting to note that no difference have been identified among CVSs carrying two different genotypes (wild type and affected by Spinal Muscolar Atrophy SMA1; OMIM#253300) further indicating that metabolic profile depends only from cell type and its differentiation stage.

The sharp distinction between hiPSCs and the other cells is confirmed by electronic nose data not only in terms of the collective behavior of the sensor array but also in terms of the individual sensors signals. The PCA scores plot in Fig. 5 shows the segregation of hiPSCs occurs along the first principal component confirming that this separation is a common trend for all the sensors of the array. For some of the sensor, instead of the trend there is a net separation between hiPSCs and the other cases (see Fig. 5). Interestingly, sensor H_2_TPP shows a smaller response to hiPSCs while the response of sensors TBPFeCl and Br_8_TPPH2 to hiPSCs is larger than to the other cases. Sensors TBPMnCl and TBPZn clearly separates from the other cases the floating EBs at day 1 and 4 respectively.

The electronic nose shows the tendency to discriminate among the different steps, from pluripotent to differentiated cells. It is important to note that only exploratory analysis have been applied in this paper. The collection of larger datasets will enable the development of classifier models for the automatic identification of samples.

The experiments here illustrated aimed at studying the differences among homogenous cell cultures but these results, and in particular the large separation between hiPSCs and the other samples, is promising about the identification of the presence of residual pluripotent cells in a mixture of differentiated cells. It is important to point out that this goal requires the extend the study to quantitative analysis of mixtures of pluripotent and differentiated cells.

## Materials and Methods

### Cell culture and differentiation

hiPSCs have been reprogrammed from residual CVS obtained from prenatal diagnosis of healthy or affected fetuses obtained between the 10th and 12th week of gestation.

Cells have been cultured in Petri dishes in standard condition of 37 °C and 5% CO_2_ atmosphere. Cell culture medium and protocols for human trophoblast cells, mouse embryonic fibroblasts (MEF), hiPSCs, *in vitro* differentiation into three germ layers and neural differentiation were reported in Spitalieri *et al*.^3^.

Briefly, for the reprogramming we used a single lentiviral “stem cell cassette,” flanked by loxP sites (hSTEMCCA-loxP), encoding four reprogramming factors (OCT4, SOX2, KLF4, and c-MYC) in a single polycistronic vector. For neural differentiation Retinoic Acid (RA), Sonic Hedgehog pathway agonist (Hh Ag 1.3, Curis Inc., Lexington, MA, USA), ciliary neurotrophic factor (CNTF) 20 ng/mL, brain-derived neurotrophic factor (BDNF) and glial cell line-derived neurotrophic factor (GDNF) 10 ng/mL were used. hSTEMCCA lentivirus production and fetal cells reprogramming were performed as previously described^3^.

This study was conducted according to the principles expressed in the Declaration of Helsinki, and was approved by the institutional review board of the Bioethical Committee of Fondazione PTV, Tor Vergata Hospital (prot. 0027655/2013).

All patients provided written informed consent for the collection of samples and subsequent analysis.

For each measurement sample, cells were seeded in parallel in two different Petri dishes in order to quantified cell number.

### Gas Chromatography Mass Spectrometer (GC/MS)

GC/MS was applied to the analysis of the VOCs released by the following five samples: Chorionic villus samples (CVSs); human induced Pluripotent Stem Cells (hiPSCs); floating embryoid bodies (from day 1 to day 11); floating embryoid bodies (from day 15 to day 18); early neural progenitors (early NPs).

For sake of comparison the following related culture media were also measured: Chang medium; hiPSCs medium; hiPSCs medium without FGF; hiPSCs medium without FGF with AR and SHH; EBs medium with 20% FBS.

Cell culture media were changed each 24 hours, and after each VOCs sampling. Then, the influence due to any chemical degradation of the culture media was the same in all samples. Pure culture media were measured after keeping the media for 24 hours in the same conditions used for cell culture.

Each measure was repeated three times in independent samples, always evaluating cell count for each measurement.

Cell headspace has been created using modified polymethylmethacrylate (PMMA) lids for Petri dishes. These caps were provided with a hole suitable to connect either a Solid Phase Micro-Extraction (SPME) fiber holder or a tube connection to the electronic nose.

Before any sampling procedure the lid was sterilized under UV light for 20 minutes.

VOCs from cell culture have been preconcentrated onto a polymeric fiber using Solid Phase Micro-Extraction technique. A 50/30 μm Divinylbenzene/Carboxen/PDMS (SUPELCO, Bellefonte, PA, USA) fiber. This is a general purpose fiber that has been previously used in similar studies^37, 38^.

The fibers were exposed to the cell headspace for 1 h. During the exposure the cultures were maintained in the incubator at a temperature of 37 °C and at a CO_2_ concentration of 5%.

Samples have been analyzed over three hour after their collection with a GC/MS (Shimadzu GCMS-QP2010, Kyoto, Japan) equipped with an EQUITY-5 capillary column (poly(5% diphenyl/95% dimethyl siloxane) phase (SUPELCO, Bellefonte, PA, USA). This is a general purpose non-polar column used in previous investigations about cells culture exhaled metabolites^18^. The column was 30 m length × 0.25 mm I.D. × 0.25 μm thickness.

The VOCs adsorbed in the SPME were desorbed from the fiber in splitless injection mode at 250 °C for 3 minutes in the GC injection port. VOCs were separated on the GC column using an initial oven temperature of 40 °C for 5 minutes, then increased by 7 °C/min to 220 °C, afterwards ramped by 15 °C/min to 300 °C that was held for 3 min (total runtime: 39 min). Ultra-high purity helium has been used as carrier gas, working in linear velocity constant mode, with a carrier gas pressure of 24.9 kPa, total flow of 5.9 mL/min, column flow of 0.7 mL/min and linear velocity of 30.2 cm/s.

The mass spectrometer was a single quadrupole analyzer in electron ionization mode and was set to record between 40 and 450 amu in the full scan mode. The temperature of transfer line and ion source was 250 °C. The detector voltage was set at 0.7 kV. GC-MS data were analyzed using the section GCMS post-run analysis of the GCMS solutions software (version 2.4, Shimadzu Corporation).

Compound names have been assigned using both NIST 127 and NIST 147 libraries. The identification of some of the identified compounds have been perfomed dissolving pure compounds in the respective culture medias at concentration of 0.1%. The measurement was performed using the same procedure outlined above.

The concentration of styrene and 1-hexenol, 2-ethyl was estimated using an external standard calibration^39^. Styrene and 1-hexenol-2-ethyl were added to the culture media diluting 1, 10, and 100 times the concentrations of 8.762 and 0.240 pptv respectively. Concentrations were calculated using the Antoine’s law parameters available at the NIST database (http://webbook.nist.gov/chemistry).

### Gas sensor array

Gas sensors were used to measure the VOCs in the headspace of the following cell samples (see Fig. 3): hiPSCs; floating embryoid bodies (day 1); floating embryoid bodies (day 4); EBs in adhesion (day 8); EBs in adhesion (day 10). As control also the following related culture media have been measured: hiPSCs medium; hiPSCs medium without FGF; EBs medium with 20% FBS.

The electronic nose was an ensemble of eleven quartz microbalance (QMB) gas sensors. In these sensors, a change of the mass (∆m) on the quartz surface results in a change of the frequency (∆f) of the electrical output signal of an oscillator circuit at which each QMB is connected. The quantities ∆m and ∆f are linearly proportional in the low-perturbation regime^40^. These QMBs had a fundamental frequency of 20 MHz, corresponding to a mass resolution of the order of a few nanograms (Fig. S1).

The sensing molecules were mostly porphyrins and corroles. The sensitizing molecules and their acronym used in the paper are shown in Table 3. Details about the preparation of porphyrins and corroles are found in the Supplementary Information File.

**Table 3:** List of the sensitive molecules applied to the sensors of the array.

1	5,10,15,20-tetrakis-(4-butyloxyphenyl) porphyrinCopper	TBPCu
2	5,10,15,20-tetrakis-(4-butyloxyphenyl) porphyrinCobal	TBPCo
3	5,10,15,20-tetrakis-(4-butyloxyphenyl) porphyrinZinc	TBPZn
4	5,10,15,20-tetrakis-(4-butyloxyphenyl) porphyrinMagnesium	TBPMg
5	5,10,15,20-tetrakis-(4-butyloxyphenyl) porphyrinManganeseChloride	TBPMnCl
6	5,10,15,20-tetrakis-(4-butyloxyphenyl) porphyrin-IronChloride	TBPFeCl
7	5,10,15,20-tetrakis-(4-butyloxyphenyl) porphyrin-TinDichloride	TBPSnCl_2_
8	2,3,7,8,12,13,17,18-octabromo-5,10,15,20-tetraphenylporphyrinH_2_	Br8TPPH_2_
9	2,3,7,8,12,13,17,18-octabromo-5,10,15,20-tetraphenylporphyrin oxoMolybdenum	Br8TPPMo
10	5,10,15-tris (3,5-dimethylphenyl) corroleCopper	TDMPCCu
11	5,10,15-tris (9-phenantryl) corrole	TPAC
12	H_2_-5,10,15,20-tetrakis-(4-butyloxyphenyl) porphyrinCopper	H_2_TPP

Thin films of sensing materials were deposited by spray coating on both the sides of quartz disks, from 10^−3^ M of porphyrins in CHCl_3_. For each sensor, the total coating resulted in a frequency shift of approximately 30 KHz. The sensors were housed in a Teflon measurement chamber having a volume of 8 mL.

Each QMB was connected to an individual oscillator circuit. Frequencies were measured by an integrated frequency counter and then stored on a computer.

The baseline of sensor signals was measured in a constant flow of ambient air filtered in a CaCl_2_ humidity trap. The cells headspaces were measured through a constant uptake of air at the constant flow of 75 sccm for three minutes. The same flow was used with reference air. Baseline was restored after each measurement.

The difference of the sensors signals in reference air and in cells-VOCs enriched air was considered as the sensor response.

### Statistical analysis

The statistical significance of GC/MS VOCs abundances and sensors signals have been evaluated with the a parametric Kruskal-Wallis rank sum test.

The absolute abundances of GC-MS identified peaks and the sensor responses were arranged in matrices and then analysed with multivariate data analysis.

Principal Component Analysis (PCA) and Partial Least Squares Discriminant Analysis (PLSDA) were used for exploration and classification purposes. PLSDA model has been cross-validated with a leave-one-out method. Classifier performance were evaluated with the classification rate, the ROC, and the area under the ROC. Confidence intervals were evaluated with a random labelling permutation test.

All data analysis was performed in Matlab.

## Electronic supplementary material

Supplementary Information
