# Supplementary material for: A preliminary analysis of volatile metabolites of human induced pluripotent stem cells along the in vitro differentiation
Source: Sci Rep. 2017 May 9;7:1621. doi: 10.1038/s41598-017-01790-5 (PMC5431616; doi:10.1038/s41598-017-01790-5)
Supplement: Supplementary file 1 — Supplementary Information [file 41598_2017_1790_MOESM1_ESM.doc]

**A preliminary analysis of volatile metabolites of human induced pluripotent stem cells along their *in vitro* differentiation**

Rosamaria Capuano^1*^, Paola Spitalieri^2*^, Rosa Valentina Talarico^2^, Ana Carolina Domakoski^3^, Alexandro Catini^1^, Roberto Paolesse^3^, Eugenio Martinelli^1^, Giuseppe Novelli^2^, Federica Sangiuolo^2§^ and Corrado Di Natale^1§^

^1^Department of Electronic Engineering, University of Rome Tor Vergata, Via del Politecnico 1, 00133 Rome, Italy

^2^Department of Biomedicine and Prevention, University of Rome Tor Vergata, Via Montpellier 1, 00133 Rome, Italy

^3^Department of Chemical Science and Technology, University of Rome Tor Vergata, Via della Ricerca Scientifica, 00133 Rome, Italy

*These authors contribute equally to this work.

§ correspondence to: dinatale@eln.uniroma2.it and sangiuolo@med.uniroma2.it

**SUPPLEMENTARY INFORMATION**

**Electronic Nose Details**

The electronic nose described in this paper is the last version of a series of instruments designed, since 1996, at the University of Rome Tor Vergata.

The core of the instrument is an array of up to twelve 20 MHz Quartz Microbalances (QMBs). Sensors are placed in a measurement cell whose volume is approximately 8 cm^3^. The gas sensors are complemented by temperature and relative humidity sensors. Each QMB is connected to an oscillator circuit, the frequencies of the oscillators outputs are measured taking advantage of temperature compensated reference quartz that allows for a frequency resolution of 0.1 Hz. Electronics is implemented in a field programmable gate-array. Gaseous samples delivery is controlled by an embedded low noise, high-precision and durable miniature diaphragm pump (0-200 sccm) and an optimized micro channel manifold made in polymethylmethacrylate shaped by a computer numerical control machine tool.

The instrument is connected to a computer and powered via a single USB connection. The electronic nose functions and data acquisition are controlled with a proprietary software running in Matlab.


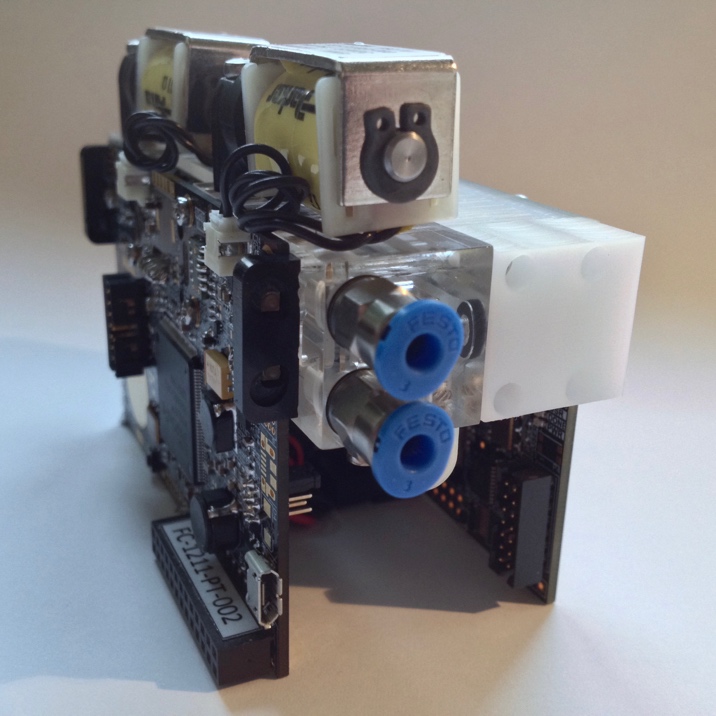


***Figure S1****: Close view of the electronic nose. On the right side the sensors cell and the board with the sensors interface. On the left side the fluidic manifold, the frequency counter and interface board. Sample and reference inlets are visible in the forefront.*

**Sensitive materials**

The list of sensitive molecules is shown in table S1. The synthesis of porphyrins and corroles have been previously reported, references are given in table S1, except TPAC whose synthesis is described below.

|  | Sensitive molecule | Acronym | Ref. |
| --- | --- | --- | --- |
| 1 | 5,10,15,20-tetrakis-(4-butyloxyphenyl)porphyrinCopper | TBPCu | 1 |
| 2 | 5,10,15,20-tetrakis-(4-butyloxyphenyl)porphyrinCobal | TBPCo | 1 |
| 3 | 5,10,15,20-tetrakis-(4-butyloxyphenyl)porphyrinZinc | TBPZn | 1 |
| 4 | 5,10,15,20-tetrakis-(4-butyloxyphenyl)porphyrinMagnesium | TBPMg | 1 |
| 5 | 5,10,15,20-tetrakis-(4-butyloxyphenyl)porphyrinManganeseChloride | TBPMnCl | 1 |
| 6 | 5,10,15,20-tetrakis-(4-butyloxyphenyl)porphyrin-IronChloride | TBPFeCl | 1 |
| 7 | 5,10,15,20-tetrakis-(4-butyloxyphenyl)porphyrin-TinDichloride | TBPSnCl2 | 1 |
| 8 | 2,3,7,8,12,13,17,18-octabromo-5,10,15,20-tetraphenylporphyrinH_2_ | Br8TPPH2 | 2 |
| 9 | 2,3,7,8,12,13,17,18-octabromo-5,10,15,20-tetraphenylporphyrin oxoMolybdenum | Br8TPPMo | 2 |
| 10 | 5,10,15-tris(3,5-dimethylphenyl)corroleCopper | TDMPCCu | 3 |
| 11 | 5,10,15-tris(9-phenantryl)corrole | TPAC |  |
| 12 | H_2_-5,10,15,20-tetrakis-(4-butyloxyphenyl)porphyrinCopper | H2TPP | 1 |

***Table S1****: list of the sensitive molecules applied to the sensors of the array.*

*References quoted in table:*

1. *Buchler, J. in The Porphyrins, ed. D. Dolphin, Academic Press, New York, 1978, vol. 1, 38*
2. *Bhyrappa, P.; Krishnan, V. Inorg. Chem., 1991, 30, 239*
3. *Brand, H.; J. Arnold, J. Coord. Chem. Rev. 1995, 140, 137.*
4. *Paolesse, R.; Marini, A.; Nardis, S.; Froiio, A.; Mandoj, F.; Nurco, D.; Prodi, L.; Montalti, M.; Smith. K. J. Porphyrins Phthalocyanines, 2003, 7, 25-36*

**Synthesis of 5,10,15-tris(9-phenantryl)corrole (TPAC)**

*Materials*

Silica gel 60 (70–230 mesh, Sigma Aldrich) are used for column chromatography. Reagents and solvents (Aldrich) are of the highest grade available and are used without further purification. ^1^H and spectrum are recorded with a Bruker AV300 spectrometer operating at 300 MHz in CDCl_3_ at 300K. Chemical shifts are given in ppm relative to residual solvent (7.26 ppm). UV-vis spectra are recorded in CH_2_Cl_2_ on a Cary 50 spectrophotometer.

*Synthesis*

Pyrrole (4.2 mL, 60 mmol) and 9-Phenanthrenecarboxaldehyde (1.29 g, 6.3 mmol), are dissolved in 20 mL of CH_2_Cl_2_, solution degassed with N_2_ for 10 minutes, then TFA (12 μL, 0.16 mmol) is added and mixture stirred at room temperature. The course of the reaction is monitored by adding DDQ to an aliquot of the reaction. After 3 hours the UV-vis spectrum suggested the corrole formation; chloranil is added (1.2 g, 4.87 mmol) and mixture further stirred for 20 minutes. Solvent is removed and residue purified by silica gel column eluted with CH_2_Cl_2_/petroleum ether 2:1. Fraction corresponding to the titled corrole was collected and crystallized by CH_2_Cl_2_/CH_3_OH. Yield 298 mg (17%). Mp > 300 °C. UV-vis (CH_2_Cl_2_): λ_max_, nm (log ε) 415 (5.03), 568 (4.27), 608 (4.08), 636 (3.87). ^1^H NMR (300 MHz, CDCl_3_): δ, ppm 9.01 (br s, 2 H), 8.98 (br s, 3 H), 8.96-8.91 (m, 3 H), 8.55-8.46 (m, 4 H), 8.40-8.38 (m, 2 H), 8.33-8.29 (m, 2 H), 8.11-8.03 (m, 3 H), 7.91-7.58 (m, 12 H), 7.49-7.31 (m, 3 H), 7.24-7.20 (m, 1 H), -1.35 (br s, 3 H). C_61_H_38_N_4_: calcd. C 88.59, H 4.63, N 6.77; found C 88.64, H 4.69, N 6.70


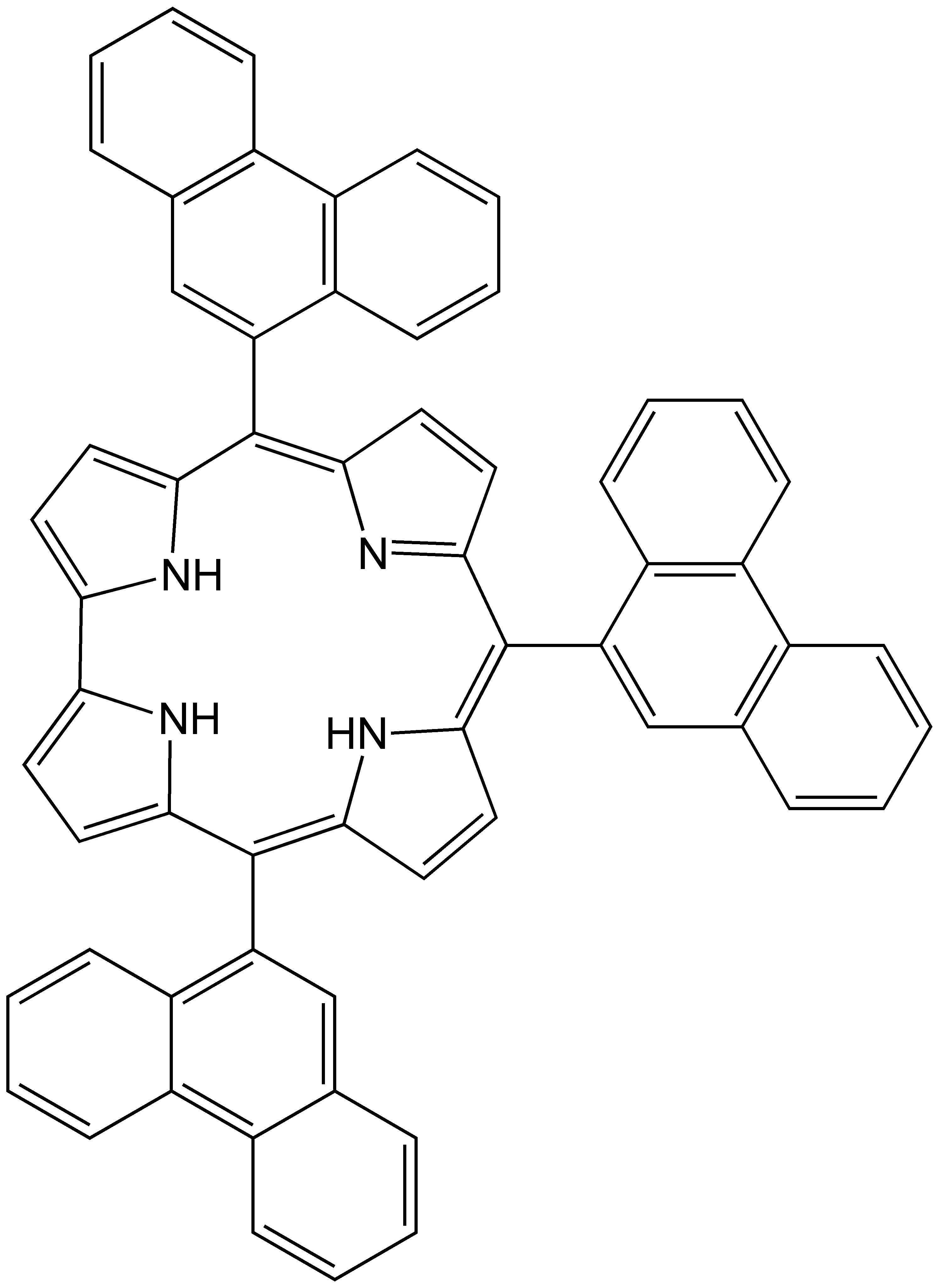


***Figure S2****: Structure of 5,10,15-tris(9-phenantryl)corrole*

**GC/MS list of identified compounds**

| **Ret. Time** | **Compound Name** | **Identification** | **CAS #** | **concentrtation** | **CVS** | **hiPSCs** | **EB**  **1-4 days** | **EB**  **9-11 days** | **EB**  **15-18 days** | **Early NPs** | **CVS medium** | **hiPSCs medium** | **hiPSCs medium without FGF** | **EB 9-11 days**  **medium** | **EBs medium with 20% FBS** |
| --- | --- | --- | --- | --- | --- | --- | --- | --- | --- | --- | --- | --- | --- | --- | --- |
| **4.023** | Acetamide, 2-fluoro- | Putative 77% | 640 - 19 - 7 |  | X | X | X | X | X | X | X | X | X | X |  |
| **4.309** | 2-Propenoic acid, 2-methyl-, methyl ester | Putative  94% | 80 - 62 - 6 |  | X | X | X | X | X | X | X | X | X | X |  |
| **4.729** | Butane, 2-ethoxy- | Putative 83% | 2679 - 87 - 0 |  |  |  |  | X | X |  |  |  |  | X |  |
| **6.725** | Hexanal | Identified | 66 - 25 - 1 |  |  |  | X |  | X |  |  |  |  |  |  |
| **9.270** | 3-Hexen-1-ol, propanoate, (Z)- | Identified | 33467 - 74 - 2 |  | X |  |  |  |  | X |  |  |  |  |  |
| **9.486** | Styrene | Identified | 629 - 20 - 9 |  | X | X | X | X | X | X | X | X | X | X | X |
| **11.342** | Acetone | Identified | 67 - 64 - 1 |  |  |  |  |  | X |  |  |  |  |  |  |
| **11.977** | Cyclobutane, 1,2,3,4-tetramethyl- | Putative 84% | 69531 - 57 - 3 |  |  |  |  | X |  |  |  |  |  |  |  |
| **12.229** | Propane, 1,1'-sulfonylbis- | Putative 79% | 598 - 03 - 8 |  | X |  |  |  |  |  |  |  |  |  |  |
| **12.362** | Ethanone, 1-cyclopropyl-2-(4-pyridinyl)- | Putative 82% | 6580 - 95 - 6 |  | X |  |  |  | X |  | X | X |  |  |  |
| **13.204** | Benzene, 1-methyl-2-(1-methylethyl)- | Identified | 527 - 84 - 4 |  | X |  |  |  |  |  | X | X |  |  |  |
| **13.345** | 1-Hexanol, 2-ethyl- | Identified | 104 - 76 - 7 |  | X | X | X | X | X | X | X | X | X | X | X |
| **14.098** | 1,4-Cyclohexadiene, 1-methyl-4-(1-methylethyl)- | Identified | 99 - 85 - 4 |  | X |  |  |  |  |  | X | X |  |  |  |
| **14.424** | Propanoic acid, 2-hydroxy-2-methyl-, ethyl ester | Identified | 80 - 55 - 7 |  | X |  |  |  |  |  |  |  |  |  |  |
| **15.178** | Nonanal | Identified | 124 - 19 - 6 |  | X | X | X | X | X | X | X | X | X |  | X |
| **15.250** | Vinyl butyrate | Identified | 123 - 20 - 6 |  |  |  |  |  | X |  |  |  |  |  |  |
| **15.474** | Ethanone, 2,2'-(octahydro-2,3-quinoxalinediylidene)bis[1-phenyl- | Putative 84% | 296244 - 70 - 7 |  | X | X | X | X | X | X | X | X |  | X | X |
| **17.404** | Tridecane | Identified | 629 - 50 - 5 |  |  | X | X |  |  |  |  |  | X |  |  |
| **17.469** | Decanal | Identified | 112 - 31 - 2 |  | X | X |  | X |  |  | X | X |  |  |  |
| **20.553** | Propanoic acid, 2-methyl-, anhydride | Identified | 97 - 72 - 3 |  | X | X |  | X |  | X | X |  | X |  |  |
| **20.958** | Butanoic acid, 2-methylpropyl ester | Identified | 539 - 90 - 2 |  | X | X | X | X |  |  | X | X | X |  |  |
| **22.777** | 1-Decene, 9-methyl- | Putative 84% | 61142 - 78 - 7 |  | X | X |  |  |  |  |  |  |  |  |  |
| **23.440** | Phenol, 3,5-bis(1,1-dimethylethyl)- | Putative 80% | 1138 - 52 - 9 |  | X | X |  |  |  |  |  |  |  |  |  |
| **24.010** | Nonane, 5-methyl-5-propyl- | Putative 87% | 17312 - 75 - 3 |  | X | X |  |  |  |  |  |  |  |  |  |
| **24.967** | Hexadecane | Identified | 17301 - 25 - 6 |  | X | X | X | X | X | X | X | X | X | X | X |
| **26.324** | Methanone, (4-aminophenyl)phenyl- | Identified | 1137 - 41 - 3 |  | X |  |  |  |  |  |  |  |  |  |  |
| **26.609** | Heptadecane | Identified | 61141 - 72 - 8 |  | X | X | X | X |  |  |  |  |  |  |  |
| **26.825** | 3,5-Dimethyl-4-octanone | Identified | 7335 - 17 - 3 |  | X | X |  |  |  |  |  |  |  |  |  |
| **27.500** | Butanoic acid, anhydride | Identified | 106 - 31 - 0 |  | X |  |  |  |  |  |  |  |  |  |  |
| **28.161** | Heptadecane | Identified | 629 - 78 - 7 |  | X | X | X | X |  | X |  |  |  |  |  |
| **28.310** | 3,5-Dimethyl-4-octanone | Identified | 7335 - 17 - 3 |  | X | X |  |  |  |  | X | X | X | X | X |
| **28.811** | 1-Tridecyn-4-ol | Putative 81% | 74646 - 37 - 0 |  | X |  |  |  |  |  |  | X |  |  |  |
| **29.163** | 1,2-Benzenedicarboxylic acid, bis(2-methylpropyl) ester | Putative 83% | 84 - 69 - 5 |  | X | X | X | X | X | X |  |  |  |  |  |

*Table* ***S2****: list of identified and putative compounds. Cross mark the samples where the compound has been found. Identified samples have been identified after a comparison of elution time and mass spectra with a reference standard. Compounds only identified via library comparison are called putative, the percentage score of identification of putatuve compounds is also indicated.*

**GC/MS Loadings plot**

**Figure S3:** *Plot of the first and second loadings of the PCA calculated on the GC/MS VOCs profiles. A magnified view of the region around the origin is shown in the inset. Loadings are labeled with a number according to the following table:*

| *1* | *Hexanal* | *10* | *Propanoic acid, 2-methyl-, anhydride* |
| --- | --- | --- | --- |
| *2* | *3-Hexen-1-ol, propanoate, (Z)-* | *11* | *Butanoic acid, 2-methylpropyl ester* |
| *3* | *Styrene* | *12* | *Phenol, 3,5-bis(1,1-dimethylethyl)-* |
| *4* | *Ethanone, 1-cyclopropyl-2-(4-pyridinyl)-* | *13* | *Propanoic acid, 2-methyl-, anhydride* |
| *5* | *1-Hexanol, 2-ethyl-* | *14* | *Hexadecane* |
| *6* | *Nonanal* | *15* | *Dodecane, 4,6-dimethyl-* |
| *7* | *Ethanone, 2,2'-(octahydro-2,3-quinoxalinediylidene)bis[1-phenyl-* | *16* | *3-Hexanone, 2,4-dimethyl* |
| *8* | *Tridecane* | *17* | *Heptadecane* |
| *9* | *Decanal* | *18* | *3,5-Dimethyl-4-octanone* |

**Styrene and 1-hexanol-2-ethyl concentration versus total cell counts.**

**
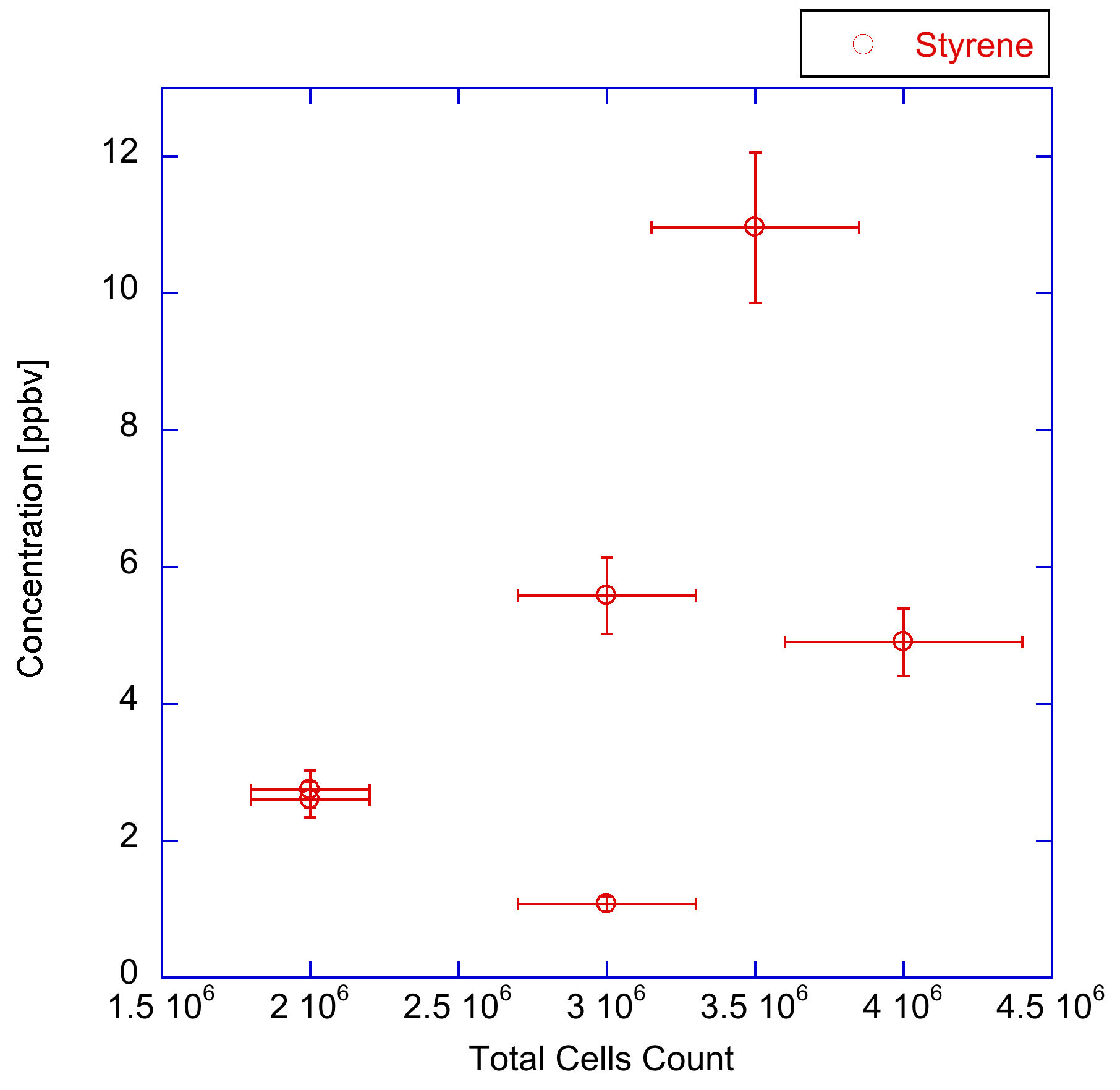

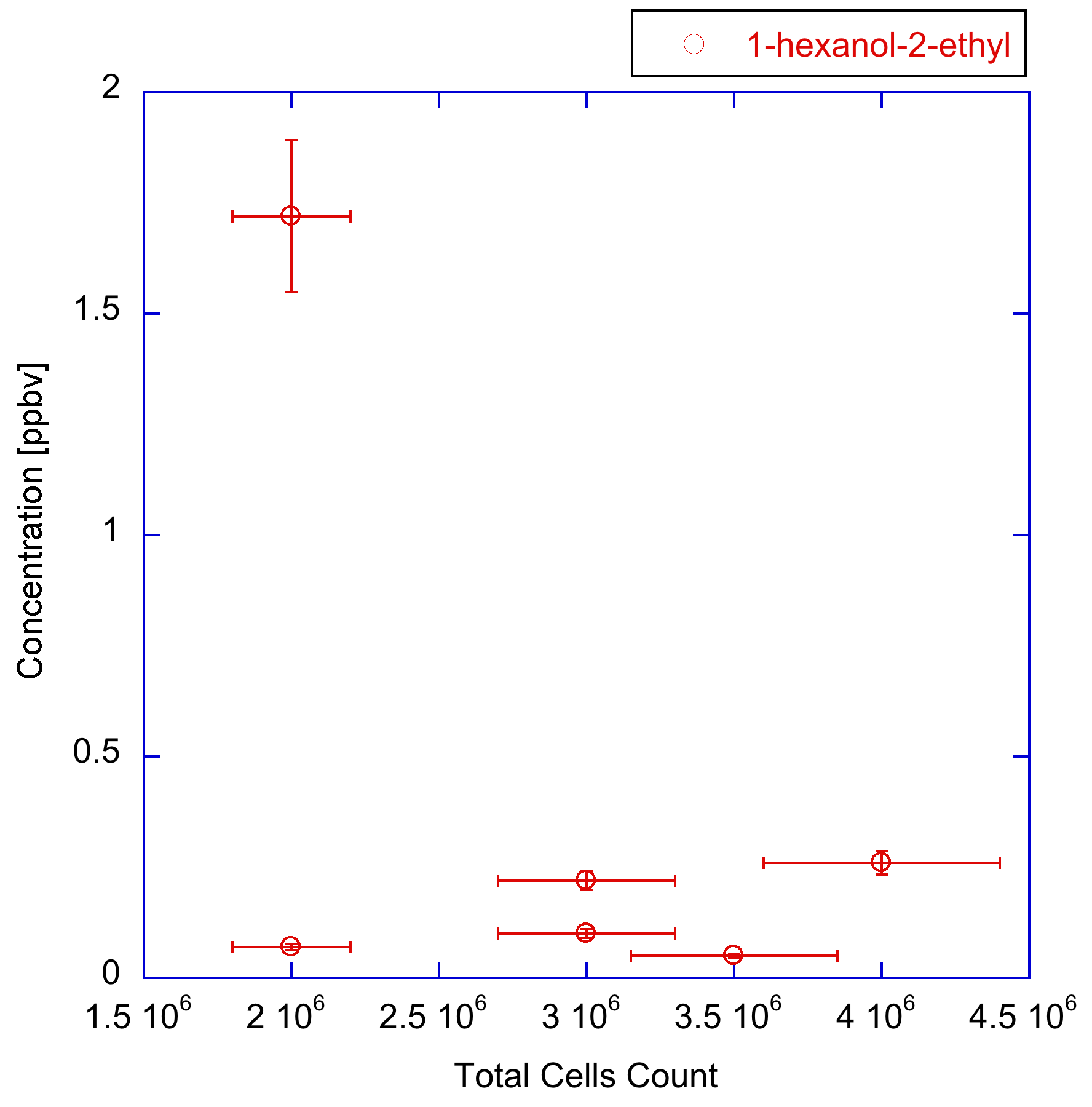
**

**Figure S4:** *Concentration of styrene and 1-hexanol-2-ethyl versus the total cells counts. Cell counts and concentrations are averaged over three samples and maximum spreads are plotted. It is interesting to note that the abundant concentration of 1-hexanol-2-ethyl in CVS does not depend on the total cells count.*

**Partial least square discriminant analysis classification model of electronic nose data**

The properties of electronic nose data to identify hiPSCs, floating EBs, and EBs in adhesion has been evaluated calculating, in Matlab, a partial least square discriminant analysis (PLSDA) model.

The model was based on six latent variables chosen with a leave-one-out procedure minimizing the root square mean error of classification.

The classifier applied to the training data results in 92.5% of correct predictions, errors are between floating EB and EB in adhesion. The confusion matrix is shown in table S2.

| *Real / predicted* | *hiPSCs* | *floating EBs* | *EBs in adhesion* |
| --- | --- | --- | --- |
| *hiPSCs* | *9* | *0* | *0* |
| *floating EBs* | *0* | *16* | *2* |
| *EBs in adhesion* | *0* | *1* | *12* |

***Table S2:*** *confusion matrix of the PLSDA model calculated on the cross-validated training data.*

The statistical significance of the model has been evaluated with a permutation test where the class membership is randomly attributed and a cross-validated PLSDA model is calculated every time[[1]](#footnote-2).

The permutation allows to evaluate the statistical properties of the hypothesis about the class membership of the dataset. Specifically, to test the statistical significance of the hypothesis that electronic nose data identify the three stages of stem cells development. The assumption of the permutation test is that most of any other randomly selected class membership obtains worse prediction with respect to the hypothesized one.

10,000 random permutations have been performed. Figure S4 shows the distribution of the classification rate. The distribution is fitted with a Gaussian probability distribution function. The results obtained by the hypothesis lies outside the 99% confidence bounds (3σ) of the distribution of the classification rate. This can be considered the confidence interval of the classification and then a statistical test about the hypothesis.

Figure S5 shows the ROC curves of the hypothesis and the permuted classifiers, and Figure S6 shows the distribution of the area under the ROC (AUROC). In all the three cases, the AUROC of the hypothesis lies beyond the 99% confidence bound.

***Figure S5:*** *Histogram shows the distribution of the classification rates calculated on 10,000 random class membership permutation. The fitted normal distribution is also plotted as a continuous line. Dashed vertical lines mark the 99% confidence bound. The classification rate obtained by the hypothesis is shown as a continuous vertical continuous line.*

***Figure S6:*** *ROC curves for each class. The curves obtained with hypothesis are plotted with a thickest line and lie on the upper left corner of each plot.*

***Figure S7:*** *Histograms show the distribution of AUROCs of the ROC curves shown in Figure S5. The fitted normal distributions are also plotted as continuous lines. Dashed vertical lines mark the 99% confidence bound. The AUROC obtained by the hypothesis are shown, in each plot, as a continuous vertical line.*

**Principal Component Analysis of all electronic nose data**

**
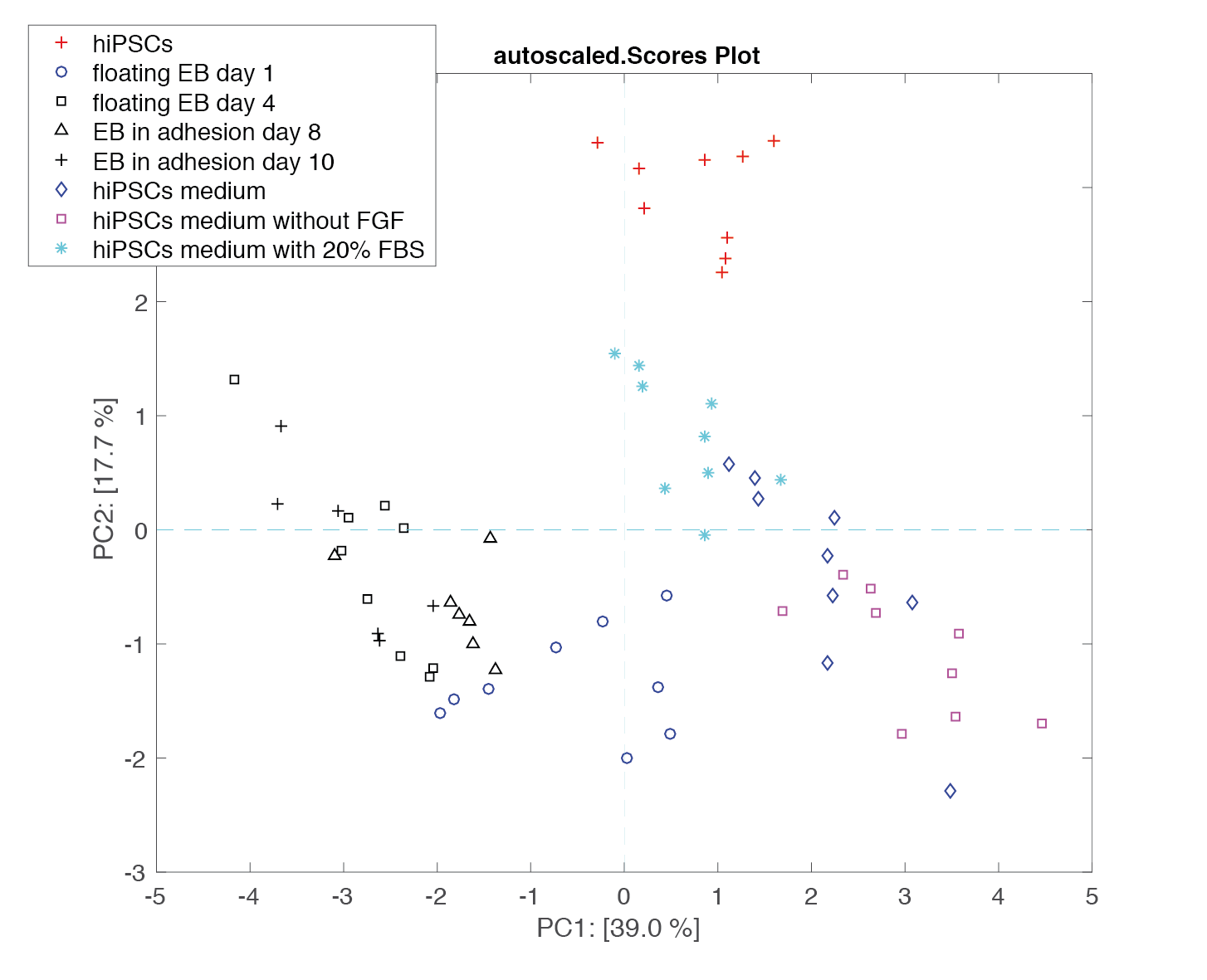
**

***Figure S8:*** *PCA scores plot of the totality of electronic nose data. The data related to culture media are separately plotted from the cells. Figure evidences the individual contribution of cells respect to the background compounds released by the culture media.*

1. *Westerhuis, J. et al. Metabolomics 2008, 4, 81-89* [↑](#footnote-ref-2)
